# Bis[3,5-difluoro-2-(4-methyl­pyridin-2-yl)phenyl-κ^2^ *C* ^1^,*N*](picolinato-κ^2^ *N*,*O*)iridium(III) chloro­form monosolvate

**DOI:** 10.1107/S1600536811030856

**Published:** 2011-08-06

**Authors:** Young-Inn Kim, Hoe-Joo Seo, Seong-Jae Yun, Young-Kwang Song, In-Chan Kim, Sung Kwon Kang

**Affiliations:** a Department of Chemistry Education and Interdisciplinary Program of Advanced Information and Display Materials, Pusan National University, Busan 609-735, Republic of Korea; b Department of Chemistry, Pusan National University, Busan 609-735, Republic of Korea; c Department of Chemistry, Chungnam National University, Daejeon 305-764, Republic of Korea

## Abstract

In the title complex, [Ir(C_12_H_8_F_2_N)_2_(C_6_H_4_NO_2_)]·CHCl_3_, two similar mol­ecules of each component comprise the asymmetric unit. The independent complex mol­ecules are linked by inter­molecular π–π inter­actions [centroid–centroid distance = 3.830 (4) Å]. The Ir^III^ ion adopts a distorted octa­hedral geometry, being coordinated by three N atoms, two C atoms, and one O atom of three bidentate ligands, with the N atoms arranged meridionally.

## Related literature

For general background to luminescent Ir complexes, see: Ulbricht *et al.* (2009 ▶); Chi & Chou (2010 ▶). For phenyl­pyridine Ir complexes, see: Lyu *et al.* (2006 ▶); Nazeeruddin *et al.* (2003 ▶); Seo *et al.* (2010 ▶); Sasabe & Kido (2011 ▶); Aoki *et al.* (2011 ▶). For phospho­rescent Ir complexes, see: Takizawa *et al.* (2006 ▶); Xu *et al.* (2009 ▶). For the Suzuki coupling reaction, see: Miyaura & Suzuki (1995 ▶).
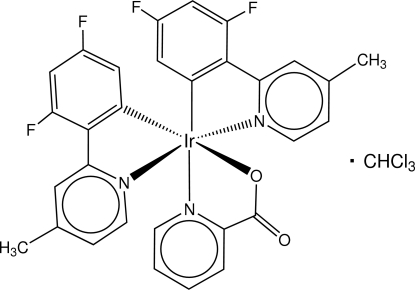


## Experimental

### 

#### Crystal data

[Ir(C_12_H_8_F_2_N)_2_(C_6_H_4_NO_2_)]·CHCl_3_
*M* *_r_* = 842.06
Triclinic, 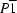

*a* = 13.421 (2) Å
*b* = 15.020 (5) Å
*c* = 16.291 (5) Å
α = 85.61 (4)°
β = 68.85 (5)°
γ = 89.26 (3)°
*V* = 3053.6 (18) Å^3^
*Z* = 4
Mo *K*α radiation
μ = 4.69 mm^−1^
*T* = 170 K
0.14 × 0.13 × 0.09 mm

#### Data collection

Bruker SMART CCD area-detector diffractometer
Absorption correction: multi-scan (*SADABS*; Bruker, 2002 ▶) *T*_min_ = 0.502, *T*_max_ = 0.665
54485 measured reflections
11349 independent reflections
9557 reflections with *I* > 2σ(*I*)
*R*_int_ = 0.038

#### Refinement

*R*[*F* ^2^ > 2σ(*F* ^2^)] = 0.033
*wR*(*F* ^2^) = 0.090
*S* = 1.03
11349 reflections
797 parameters
H-atom parameters constrained
Δρ_max_ = 2.76 e Å^−3^
Δρ_min_ = −1.28 e Å^−3^

### 

Data collection: *SMART* (Bruker, 2002 ▶); cell refinement: *SAINT* (Bruker, 2002 ▶); data reduction: *SAINT*; program(s) used to solve structure: *SHELXS97* (Sheldrick, 2008 ▶); program(s) used to refine structure: *SHELXL97* (Sheldrick, 2008 ▶); molecular graphics: *ORTEP-3 for Windows* (Farrugia, 1997 ▶); software used to prepare material for publication: *WinGX* (Farrugia, 1999 ▶).

## Supplementary Material

Crystal structure: contains datablock(s) global, I. DOI: 10.1107/S1600536811030856/tk2773sup1.cif

Structure factors: contains datablock(s) I. DOI: 10.1107/S1600536811030856/tk2773Isup2.hkl

Additional supplementary materials:  crystallographic information; 3D view; checkCIF report

## Figures and Tables

**Table:** 

Ir1—C23	1.987 (5)
Ir1—C8	1.995 (6)
Ir1—N16	2.027 (5)
Ir1—N1	2.040 (4)
Ir1—N31	2.123 (5)
Ir1—O38	2.153 (4)
Ir2—C62	1.979 (5)
Ir2—C47	1.991 (5)
Ir2—N55	2.019 (4)
Ir2—N40	2.046 (4)
Ir2—N70	2.125 (5)
Ir2—O77	2.148 (4)

**Table:** 

C23—Ir1—N16	80.4 (2)
C8—Ir1—N1	80.2 (2)
C23—Ir1—N31	97.73 (19)
C8—Ir1—O38	99.14 (19)
N31—Ir1—O38	76.70 (16)
C62—Ir2—N55	80.3 (2)
C47—Ir2—N40	79.8 (2)
N40—Ir2—N70	97.20 (17)
C47—Ir2—O77	98.49 (18)
N70—Ir2—O77	76.80 (16)

**Fig. 1.**
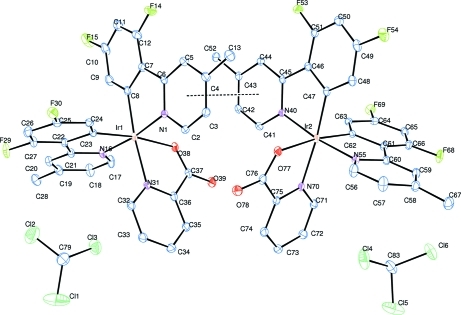
Molecular structures of the four independent molecules in (I), showing the atom-numbering scheme and 30% probability ellipsoids. The complex molecules are linked by π-π interactions (dashed lines). H atoms have been omitted for clarity.

## supplementary crystallographic information

### Comment

There has been a growing interest in luminescent iridium complexes (Ulbricht *et al.*, 2009) because of their high quantum efficiency and tunable emission energy (Chi & Chou, 2010). Especially, 2-phenylpyridine-based cyclometallated iridium(III) complexes have been reported (Lyu *et al.*, 2006; Nazeeruddin *et al.*, 2003; Seo *et al.*, 2010; Sasabe *et al.*, 2011; Aoki *et al.*, 2011) and proved to be excellent candidates for organic light-emitting diodes (OLEDs) in full color display by doping red, green to blue iridium(III) phosphors in host matrix. But pure blue emissive materials with high phosphorescence efficiency are still rare comparing to red and green ones. Recently, blue phosphorescent iridium(III) complexes bearing 2-(fluoro substituted phenyl)-4-methylpyridine were reported (Takizawa *et al.*, 2006; Xu *et al.*, 2009) and their photophysical properties were discussed. Herein, we prepared a blue emissive titled complex and its structure is reported.

In (I), two similar complex molecules and two chloroform comprise the asymmetric unit, which are linked by the intermolecular π-π interactions (centroid-centroid distance = 3.830 (4) Å) between the aromatic rings of the discrete units (Fig. 1 and Table 1). The Ir^III^ ion adopts a distorted octahedral geometry, being coordinated by three N atoms, two C atoms, and one O atom of three bidentate ligands. The angles around Ir atoms are in the range of 76.70 (16) – 99.14 (19) °. The Ir—C bond distances of 1.979 (5) – 1.995 (6) Å are shorter than the Ir—N distances of 2.019 (4) -2.125 (5) Å due to the stronger *trans* influence of the phenyl ring compared to the pyridine ring (Table 1). The N atoms of each dfpmpy ligand adopt a meridional arrangement.

### Experimental

Synthesis of 2-(2,4-difluorophenyl)-4-methylpyridine (dfpmpy): dfpmpy was prepared by Suzuki coupling reaction using 2,4-difluorophenylboronic acid and the appropriate 2-bromo-4-methylpyridine(Miyaura & Suzuki, 1995). 2-Bromo-4-methylpyridine, 2,4-difluorophenylboronic acid and tetrakis(triphenylphosphine)palladium(0) were dissolved to 50 ml of THF. After 30 ml of aqueous 2*M* Na_2_CO_3_ was delivered, the reaction mixture was heated at 343 K for 24 h. The crude product was flash chromatographed on silica gel using n-hexane/ethyl acetate as an eluent.

Synthesis of title complex: Cyclometallated iridium(III) *µ*-chloro-bridged dimer, [(dfpmpy)_2_Ir(*µ*-Cl)]_2_, was prepared from the reaction of IrCl_3_ 3H_2_O with dfpmpy in a 3:1 mixture of 2-ethoxyethanol and water at 398 K for 24 h. The dimeric iridium(III) complex, sodium carbonate and picolinic acid were dissolved 2-ethoxyethanol, and the mixture was heated at 403 K for 24 h. The mixture extracted with dichloromethane and dried over anhydrous magnesium sulfate. The crude product was flash chromatographed on silica gel using dichloromethane/methanol as an eluent. The yellow crystals were grown from its ethanol/chloroform solution by slow evaporation at room temperature.

### Refinement

All H atoms were positioned geometrically and refined using a riding model, with C—H = 0.93 - 0.98 Å, and with *U*_iso_(H) = 1.2*U*_eq_(C) for aromatic- and chloroform-H atoms, and 1.5*U*_eq_(C) for methyl-H atoms. The maximum and minimum residual electron density peaks were located at 0.84 and 0.87 Å from the Ir2 and Cl1 atoms, respectively.

### Crystal data

**Table:**

[Ir(C_12_H_8_F_2_N)_2_(C_6_H_4_NO_2_)]·CHCl_3_	*Z* = 4
*M**_r_* = 842.06	*F*(000) = 1632
Triclinic, *P*1	*D*_x_ = 1.832 Mg m^−^^3^
Hall symbol: -P 1	Mo *K*α radiation, λ = 0.71073 Å
*a* = 13.421 (2) Å	Cell parameters from 8556 reflections
*b* = 15.020 (5) Å	θ = 2.4–28.2°
*c* = 16.291 (5) Å	µ = 4.69 mm^−^^1^
α = 85.61 (4)°	*T* = 170 K
β = 68.85 (5)°	Block, yellow
γ = 89.26 (3)°	0.14 × 0.13 × 0.09 mm
*V* = 3053.6 (18) Å^3^	

### Data collection

**Table:**

Bruker SMART CCD area-detector diffractometer	9557 reflections with *I* > 2σ(*I*)
φ and ω scans	*R*_int_ = 0.038
Absorption correction: multi-scan (*SADABS*; Bruker, 2002)	θ_max_ = 25.5°, θ_min_ = 1.6°
*T*_min_ = 0.502, *T*_max_ = 0.665	*h* = −16→16
54485 measured reflections	*k* = −18→18
11349 independent reflections	*l* = −19→19

### Refinement

**Table:**

Refinement on *F*^2^	0 restraints
Least-squares matrix: full	H-atom parameters constrained
*R*[*F*^2^ > 2σ(*F*^2^)] = 0.033	*w* = 1/[σ^2^(*F*_o_^2^) + (0.0433*P*)^2^ + 10.7327*P*] where *P* = (*F*_o_^2^ + 2*F*_c_^2^)/3
*wR*(*F*^2^) = 0.090	(Δ/σ)_max_ = 0.002
*S* = 1.03	Δρ_max_ = 2.76 e Å^−^^3^
11349 reflections	Δρ_min_ = −1.28 e Å^−^^3^
797 parameters	

### Special details

**Table:**

Geometry. All e.s.d.'s (except the e.s.d. in the dihedral angle between two l.s. planes) are estimated using the full covariance matrix. The cell e.s.d.'s are taken into account individually in the estimation of e.s.d.'s in distances, angles and torsion angles; correlations between e.s.d.'s in cell parameters are only used when they are defined by crystal symmetry. An approximate (isotropic) treatment of cell e.s.d.'s is used for estimating e.s.d.'s involving l.s. planes.

### Fractional atomic coordinates and isotropic or equivalent isotropic displacement parameters (Å^2^)

**Table:**

	*x*	*y*	*z*	*U*_iso_*/*U*_eq_	
Ir1	0.401921 (17)	0.127006 (13)	0.272846 (13)	0.03178 (7)	
N1	0.2687 (3)	0.1283 (3)	0.3844 (3)	0.0308 (9)	
C2	0.2679 (4)	0.1269 (4)	0.4673 (4)	0.0362 (12)	
H2	0.333	0.1265	0.4755	0.043*	
C3	0.1764 (5)	0.1260 (4)	0.5397 (4)	0.0410 (13)	
H3	0.1797	0.1238	0.5959	0.049*	
C4	0.0787 (5)	0.1285 (4)	0.5295 (4)	0.0435 (14)	
C5	0.0793 (5)	0.1313 (4)	0.4446 (4)	0.0442 (14)	
H5	0.0146	0.1332	0.4357	0.053*	
C6	0.1727 (4)	0.1316 (4)	0.3727 (4)	0.0349 (12)	
C7	0.1850 (5)	0.1371 (4)	0.2791 (4)	0.0385 (13)	
C8	0.2903 (5)	0.1417 (4)	0.2189 (4)	0.0376 (13)	
C9	0.3059 (6)	0.1527 (4)	0.1297 (4)	0.0522 (17)	
H9	0.3747	0.1585	0.0879	0.063*	
C10	0.2206 (7)	0.1552 (5)	0.1035 (5)	0.064 (2)	
C11	0.1183 (7)	0.1485 (5)	0.1598 (5)	0.065 (2)	
H11	0.0615	0.1497	0.1399	0.078*	
C12	0.1027 (5)	0.1400 (5)	0.2474 (5)	0.0528 (17)	
C13	−0.0236 (5)	0.1297 (5)	0.6073 (5)	0.0603 (19)	
H13A	−0.0779	0.1572	0.589	0.09*	
H13B	−0.0131	0.1632	0.6515	0.09*	
H13C	−0.0452	0.0697	0.6312	0.09*	
F14	0.0006 (3)	0.1328 (3)	0.3045 (3)	0.0715 (12)	
F15	0.2387 (4)	0.1630 (4)	0.0157 (3)	0.0980 (19)	
N16	0.5221 (4)	0.1191 (3)	0.1547 (3)	0.0371 (11)	
C17	0.5798 (6)	0.1900 (4)	0.1074 (4)	0.0537 (17)	
H17	0.5673	0.2454	0.1308	0.064*	
C18	0.6564 (6)	0.1834 (4)	0.0259 (4)	0.0582 (19)	
H18	0.6966	0.2335	−0.0046	0.07*	
C19	0.6744 (5)	0.1021 (5)	−0.0113 (4)	0.0497 (16)	
C20	0.6134 (5)	0.0297 (4)	0.0377 (4)	0.0441 (14)	
H20	0.6232	−0.0257	0.0142	0.053*	
C21	0.5383 (5)	0.0381 (4)	0.1208 (4)	0.0361 (12)	
C22	0.4690 (4)	−0.0320 (4)	0.1802 (4)	0.0343 (12)	
C23	0.3943 (4)	−0.0029 (3)	0.2598 (4)	0.0317 (11)	
C24	0.3277 (4)	−0.0667 (4)	0.3210 (4)	0.0347 (12)	
H24	0.2779	−0.0497	0.3737	0.042*	
C25	0.3361 (4)	−0.1545 (4)	0.3030 (4)	0.0377 (13)	
C26	0.4064 (5)	−0.1847 (4)	0.2266 (4)	0.0457 (15)	
H26	0.4093	−0.2448	0.2158	0.055*	
C27	0.4721 (5)	−0.1219 (4)	0.1668 (4)	0.0433 (14)	
C28	0.7599 (6)	0.0929 (5)	−0.1003 (4)	0.0618 (19)	
H28A	0.7788	0.1508	−0.1313	0.093*	
H28B	0.7337	0.0557	−0.1337	0.093*	
H28C	0.8218	0.0663	−0.0928	0.093*	
F29	0.5416 (3)	−0.1517 (2)	0.0920 (3)	0.0639 (11)	
F30	0.2726 (3)	−0.2159 (2)	0.3643 (3)	0.0501 (9)	
N31	0.5127 (3)	0.1215 (3)	0.3388 (3)	0.0306 (10)	
C32	0.5541 (4)	0.0478 (4)	0.3645 (4)	0.0368 (13)	
H32	0.5367	−0.0076	0.3511	0.044*	
C33	0.6221 (5)	0.0523 (4)	0.4105 (4)	0.0450 (14)	
H33	0.65	0.0003	0.4279	0.054*	
C34	0.6486 (5)	0.1338 (4)	0.4307 (5)	0.0468 (15)	
H34	0.6931	0.1377	0.4628	0.056*	
C35	0.6076 (4)	0.2094 (4)	0.4025 (4)	0.0422 (14)	
H35	0.6256	0.2654	0.4141	0.051*	
C36	0.5403 (4)	0.2019 (4)	0.3572 (4)	0.0349 (12)	
C37	0.4926 (4)	0.2819 (4)	0.3238 (4)	0.0368 (13)	
O38	0.4333 (3)	0.2655 (2)	0.2815 (3)	0.0375 (9)	
O39	0.5156 (3)	0.3572 (3)	0.3383 (3)	0.0510 (11)	
Ir2	0.178581 (16)	0.386356 (13)	0.679992 (13)	0.02913 (7)	
N40	0.1713 (3)	0.3834 (3)	0.5569 (3)	0.0268 (9)	
C41	0.2550 (4)	0.3808 (4)	0.4814 (3)	0.0335 (12)	
H41	0.323	0.3798	0.4842	0.04*	
C42	0.2466 (4)	0.3797 (4)	0.4009 (4)	0.0346 (12)	
H42	0.3078	0.3786	0.3504	0.042*	
C43	0.1472 (4)	0.3803 (4)	0.3943 (4)	0.0336 (12)	
C44	0.0600 (4)	0.3812 (4)	0.4718 (3)	0.0328 (12)	
H44	−0.0084	0.3807	0.4698	0.039*	
C45	0.0721 (4)	0.3830 (3)	0.5524 (3)	0.0291 (11)	
C46	−0.0133 (4)	0.3818 (3)	0.6390 (3)	0.0310 (11)	
C47	0.0211 (4)	0.3779 (3)	0.7112 (3)	0.0320 (11)	
C48	−0.0575 (5)	0.3710 (4)	0.7972 (4)	0.0379 (13)	
H48	−0.038	0.3674	0.8466	0.045*	
C49	−0.1628 (5)	0.3698 (4)	0.8062 (4)	0.0435 (15)	
C50	−0.1982 (5)	0.3752 (4)	0.7375 (4)	0.0421 (14)	
H50	−0.2707	0.3746	0.7465	0.051*	
C51	−0.1219 (4)	0.3814 (4)	0.6550 (4)	0.0356 (12)	
C52	0.1340 (5)	0.3769 (4)	0.3067 (3)	0.0411 (13)	
H52A	0.1172	0.4353	0.2875	0.062*	
H52B	0.0771	0.3359	0.3128	0.062*	
H52C	0.1993	0.3575	0.264	0.062*	
F53	−0.1575 (2)	0.3876 (3)	0.5867 (2)	0.0478 (9)	
F54	−0.2367 (3)	0.3645 (3)	0.8902 (2)	0.0608 (11)	
N55	0.1675 (4)	0.3956 (3)	0.8060 (3)	0.0356 (10)	
C56	0.1626 (6)	0.3235 (4)	0.8620 (4)	0.0542 (18)	
H56	0.1684	0.2669	0.8415	0.065*	
C57	0.1492 (7)	0.3319 (5)	0.9481 (4)	0.061 (2)	
H57	0.1467	0.2809	0.985	0.073*	
C58	0.1394 (5)	0.4150 (4)	0.9813 (4)	0.0456 (15)	
C59	0.1410 (5)	0.4881 (4)	0.9246 (4)	0.0419 (14)	
H59	0.133	0.5449	0.9451	0.05*	
C60	0.1545 (4)	0.4783 (4)	0.8368 (4)	0.0347 (12)	
C61	0.1553 (4)	0.5487 (3)	0.7692 (3)	0.0317 (11)	
C62	0.1638 (4)	0.5172 (3)	0.6865 (3)	0.0283 (11)	
C63	0.1660 (4)	0.5807 (3)	0.6188 (3)	0.0307 (11)	
H63	0.1737	0.5629	0.5634	0.037*	
C64	0.1567 (4)	0.6694 (4)	0.6339 (4)	0.0357 (12)	
C65	0.1465 (4)	0.7018 (4)	0.7143 (4)	0.0383 (13)	
H65	0.1395	0.7624	0.7232	0.046*	
C66	0.1475 (5)	0.6394 (4)	0.7791 (4)	0.0385 (13)	
C67	0.1316 (6)	0.4252 (5)	1.0748 (4)	0.0583 (18)	
H67A	0.2006	0.441	1.075	0.088*	
H67B	0.1075	0.3698	1.1096	0.088*	
H67C	0.0818	0.4712	1.0992	0.088*	
F68	0.1375 (3)	0.6708 (2)	0.8579 (2)	0.0564 (10)	
F69	0.1581 (3)	0.7303 (2)	0.5682 (2)	0.0477 (8)	
N70	0.3477 (4)	0.3839 (3)	0.6406 (3)	0.0310 (10)	
C71	0.4140 (5)	0.4543 (4)	0.6265 (4)	0.0366 (12)	
H71	0.3859	0.5114	0.6319	0.044*	
C72	0.5216 (5)	0.4444 (4)	0.6044 (4)	0.0420 (14)	
H72	0.5657	0.4944	0.5953	0.05*	
C73	0.5651 (5)	0.3603 (4)	0.5958 (4)	0.0425 (14)	
H73	0.6383	0.3528	0.5801	0.051*	
C74	0.4968 (5)	0.2875 (4)	0.6110 (4)	0.0420 (14)	
H74	0.5236	0.23	0.606	0.05*	
C75	0.3891 (4)	0.3013 (4)	0.6337 (3)	0.0331 (12)	
C76	0.3098 (5)	0.2256 (4)	0.6528 (4)	0.0387 (13)	
O77	0.2124 (3)	0.2462 (2)	0.6793 (3)	0.0362 (9)	
O78	0.3444 (4)	0.1485 (3)	0.6427 (3)	0.0525 (12)	
C79	0.8189 (8)	−0.0330 (6)	0.1973 (6)	0.081 (3)	
H79	0.7683	−0.0532	0.2561	0.097*	
Cl1	0.9438 (3)	−0.0490 (3)	0.1971 (3)	0.185 (2)	
Cl2	0.7860 (4)	−0.0820 (2)	0.1244 (3)	0.1664 (19)	
Cl3	0.8030 (2)	0.08722 (18)	0.1801 (2)	0.1093 (9)	
C83	0.4763 (7)	0.5241 (6)	0.8416 (5)	0.074 (2)	
H83	0.4798	0.5549	0.7854	0.089*	
Cl4	0.4398 (3)	0.41077 (18)	0.84317 (19)	0.1220 (12)	
Cl5	0.5977 (2)	0.5333 (3)	0.8509 (3)	0.1506 (16)	
Cl6	0.37928 (17)	0.57276 (14)	0.92568 (17)	0.0890 (8)	

### Atomic displacement parameters (Å^2^)

**Table:**

	*U*^11^	*U*^22^	*U*^33^	*U*^12^	*U*^13^	*U*^23^
Ir1	0.03765 (12)	0.02222 (11)	0.03306 (12)	0.00448 (8)	−0.00911 (9)	−0.00650 (8)
N1	0.032 (2)	0.025 (2)	0.036 (2)	0.0037 (18)	−0.0122 (19)	−0.0071 (18)
C2	0.031 (3)	0.037 (3)	0.042 (3)	−0.001 (2)	−0.013 (2)	−0.012 (2)
C3	0.040 (3)	0.045 (3)	0.037 (3)	−0.001 (3)	−0.011 (3)	−0.013 (3)
C4	0.036 (3)	0.042 (3)	0.049 (4)	0.001 (3)	−0.009 (3)	−0.011 (3)
C5	0.038 (3)	0.043 (4)	0.053 (4)	0.009 (3)	−0.018 (3)	−0.011 (3)
C6	0.038 (3)	0.026 (3)	0.044 (3)	0.010 (2)	−0.018 (3)	−0.012 (2)
C7	0.050 (3)	0.032 (3)	0.042 (3)	0.016 (3)	−0.026 (3)	−0.008 (2)
C8	0.048 (3)	0.027 (3)	0.040 (3)	0.012 (2)	−0.017 (3)	−0.010 (2)
C9	0.067 (4)	0.047 (4)	0.044 (4)	0.026 (3)	−0.022 (3)	−0.006 (3)
C10	0.090 (6)	0.067 (5)	0.044 (4)	0.050 (4)	−0.034 (4)	−0.013 (3)
C11	0.080 (5)	0.072 (5)	0.062 (5)	0.041 (4)	−0.046 (4)	−0.019 (4)
C12	0.050 (4)	0.052 (4)	0.063 (4)	0.021 (3)	−0.028 (3)	−0.010 (3)
C13	0.043 (4)	0.081 (5)	0.050 (4)	0.004 (3)	−0.006 (3)	−0.016 (4)
F14	0.054 (2)	0.099 (4)	0.076 (3)	0.026 (2)	−0.039 (2)	−0.019 (2)
F15	0.120 (4)	0.141 (5)	0.047 (2)	0.079 (4)	−0.048 (3)	−0.020 (3)
N16	0.045 (3)	0.026 (2)	0.033 (2)	0.005 (2)	−0.005 (2)	−0.0022 (19)
C17	0.073 (5)	0.026 (3)	0.048 (4)	0.000 (3)	−0.003 (3)	−0.005 (3)
C18	0.073 (5)	0.033 (3)	0.046 (4)	−0.003 (3)	0.003 (3)	0.004 (3)
C19	0.054 (4)	0.049 (4)	0.038 (3)	0.012 (3)	−0.007 (3)	−0.003 (3)
C20	0.050 (4)	0.036 (3)	0.043 (3)	0.010 (3)	−0.012 (3)	−0.010 (3)
C21	0.044 (3)	0.028 (3)	0.035 (3)	0.007 (2)	−0.013 (2)	−0.004 (2)
C22	0.042 (3)	0.026 (3)	0.036 (3)	0.006 (2)	−0.014 (2)	−0.008 (2)
C23	0.035 (3)	0.025 (3)	0.039 (3)	0.006 (2)	−0.018 (2)	−0.005 (2)
C24	0.033 (3)	0.033 (3)	0.041 (3)	0.003 (2)	−0.017 (2)	−0.003 (2)
C25	0.036 (3)	0.029 (3)	0.050 (3)	0.001 (2)	−0.018 (3)	−0.002 (2)
C26	0.056 (4)	0.027 (3)	0.059 (4)	−0.001 (3)	−0.026 (3)	−0.006 (3)
C27	0.051 (4)	0.034 (3)	0.042 (3)	0.007 (3)	−0.012 (3)	−0.013 (3)
C28	0.058 (4)	0.069 (5)	0.042 (4)	0.013 (4)	0.002 (3)	−0.007 (3)
F29	0.085 (3)	0.034 (2)	0.054 (2)	0.0088 (19)	0.000 (2)	−0.0191 (17)
F30	0.0468 (19)	0.0295 (18)	0.067 (2)	−0.0055 (15)	−0.0135 (18)	0.0043 (16)
N31	0.026 (2)	0.026 (2)	0.036 (2)	0.0017 (17)	−0.0054 (19)	−0.0080 (18)
C32	0.033 (3)	0.027 (3)	0.046 (3)	0.000 (2)	−0.008 (2)	−0.005 (2)
C33	0.044 (3)	0.033 (3)	0.059 (4)	0.003 (3)	−0.020 (3)	−0.007 (3)
C34	0.043 (3)	0.041 (4)	0.063 (4)	0.000 (3)	−0.025 (3)	−0.007 (3)
C35	0.035 (3)	0.035 (3)	0.055 (4)	−0.003 (2)	−0.013 (3)	−0.013 (3)
C36	0.032 (3)	0.027 (3)	0.038 (3)	−0.002 (2)	−0.002 (2)	−0.008 (2)
C37	0.036 (3)	0.028 (3)	0.039 (3)	0.001 (2)	−0.003 (2)	−0.006 (2)
O38	0.045 (2)	0.0223 (19)	0.044 (2)	0.0054 (16)	−0.0135 (19)	−0.0068 (16)
O39	0.054 (3)	0.025 (2)	0.075 (3)	0.0007 (18)	−0.023 (2)	−0.013 (2)
Ir2	0.03736 (12)	0.02252 (11)	0.02688 (11)	0.00286 (8)	−0.01019 (9)	−0.00546 (8)
N40	0.028 (2)	0.024 (2)	0.027 (2)	0.0011 (17)	−0.0083 (18)	−0.0061 (17)
C41	0.027 (3)	0.038 (3)	0.033 (3)	0.005 (2)	−0.007 (2)	−0.006 (2)
C42	0.034 (3)	0.035 (3)	0.030 (3)	0.007 (2)	−0.006 (2)	−0.008 (2)
C43	0.037 (3)	0.029 (3)	0.033 (3)	0.002 (2)	−0.009 (2)	−0.008 (2)
C44	0.028 (3)	0.035 (3)	0.034 (3)	−0.002 (2)	−0.009 (2)	−0.006 (2)
C45	0.033 (3)	0.023 (3)	0.031 (3)	0.002 (2)	−0.009 (2)	−0.007 (2)
C46	0.033 (3)	0.024 (3)	0.033 (3)	−0.001 (2)	−0.006 (2)	−0.005 (2)
C47	0.037 (3)	0.025 (3)	0.030 (3)	−0.001 (2)	−0.007 (2)	−0.006 (2)
C48	0.046 (3)	0.036 (3)	0.024 (3)	0.000 (2)	−0.004 (2)	−0.001 (2)
C49	0.046 (3)	0.034 (3)	0.033 (3)	−0.006 (3)	0.006 (3)	−0.001 (2)
C50	0.032 (3)	0.042 (3)	0.041 (3)	−0.009 (2)	0.000 (3)	−0.005 (3)
C51	0.037 (3)	0.031 (3)	0.036 (3)	−0.004 (2)	−0.008 (2)	−0.009 (2)
C52	0.044 (3)	0.049 (4)	0.029 (3)	0.002 (3)	−0.012 (3)	−0.008 (3)
F53	0.0309 (17)	0.065 (2)	0.046 (2)	−0.0045 (16)	−0.0109 (15)	−0.0087 (17)
F54	0.052 (2)	0.069 (3)	0.037 (2)	−0.0077 (19)	0.0119 (17)	−0.0018 (18)
N55	0.048 (3)	0.030 (2)	0.030 (2)	0.002 (2)	−0.015 (2)	−0.0056 (19)
C56	0.092 (5)	0.033 (3)	0.042 (4)	0.016 (3)	−0.029 (4)	−0.005 (3)
C57	0.107 (6)	0.042 (4)	0.033 (3)	0.015 (4)	−0.027 (4)	0.004 (3)
C58	0.057 (4)	0.048 (4)	0.035 (3)	0.011 (3)	−0.020 (3)	−0.009 (3)
C59	0.051 (3)	0.037 (3)	0.038 (3)	0.010 (3)	−0.016 (3)	−0.013 (3)
C60	0.038 (3)	0.032 (3)	0.034 (3)	0.007 (2)	−0.014 (2)	−0.007 (2)
C61	0.038 (3)	0.027 (3)	0.031 (3)	0.003 (2)	−0.014 (2)	−0.005 (2)
C62	0.025 (2)	0.026 (3)	0.033 (3)	0.002 (2)	−0.010 (2)	−0.006 (2)
C63	0.031 (3)	0.030 (3)	0.033 (3)	0.001 (2)	−0.013 (2)	−0.004 (2)
C64	0.034 (3)	0.028 (3)	0.047 (3)	−0.002 (2)	−0.019 (3)	0.004 (2)
C65	0.041 (3)	0.024 (3)	0.049 (3)	0.007 (2)	−0.013 (3)	−0.012 (2)
C66	0.046 (3)	0.033 (3)	0.039 (3)	0.005 (2)	−0.018 (3)	−0.012 (2)
C67	0.076 (5)	0.066 (5)	0.034 (3)	0.010 (4)	−0.021 (3)	−0.005 (3)
F68	0.097 (3)	0.0344 (19)	0.044 (2)	0.0106 (19)	−0.031 (2)	−0.0183 (16)
F69	0.065 (2)	0.0322 (18)	0.049 (2)	−0.0002 (16)	−0.0263 (18)	0.0072 (15)
N70	0.041 (2)	0.025 (2)	0.031 (2)	0.0046 (19)	−0.017 (2)	−0.0096 (18)
C71	0.046 (3)	0.029 (3)	0.037 (3)	0.003 (2)	−0.017 (3)	−0.005 (2)
C72	0.044 (3)	0.037 (3)	0.045 (3)	−0.001 (3)	−0.016 (3)	−0.004 (3)
C73	0.042 (3)	0.039 (3)	0.051 (4)	0.006 (3)	−0.021 (3)	−0.009 (3)
C74	0.052 (4)	0.036 (3)	0.044 (3)	0.011 (3)	−0.024 (3)	−0.011 (3)
C75	0.046 (3)	0.028 (3)	0.030 (3)	0.008 (2)	−0.019 (2)	−0.007 (2)
C76	0.057 (4)	0.025 (3)	0.042 (3)	0.005 (3)	−0.027 (3)	−0.007 (2)
O77	0.045 (2)	0.0233 (19)	0.043 (2)	0.0042 (16)	−0.0189 (18)	−0.0066 (16)
O78	0.058 (3)	0.022 (2)	0.088 (3)	0.0081 (19)	−0.038 (3)	−0.011 (2)
C79	0.091 (6)	0.090 (7)	0.063 (5)	−0.001 (5)	−0.031 (5)	−0.002 (5)
Cl1	0.087 (2)	0.228 (5)	0.236 (5)	−0.040 (2)	−0.076 (3)	0.114 (4)
Cl2	0.284 (5)	0.097 (2)	0.201 (4)	0.040 (3)	−0.184 (4)	−0.033 (2)
Cl3	0.110 (2)	0.0724 (16)	0.135 (2)	0.0002 (14)	−0.0281 (18)	−0.0211 (16)
C83	0.078 (5)	0.077 (6)	0.052 (4)	0.034 (4)	−0.009 (4)	0.002 (4)
Cl4	0.196 (3)	0.0697 (16)	0.0861 (17)	0.0454 (18)	−0.0315 (19)	−0.0221 (13)
Cl5	0.0673 (16)	0.206 (4)	0.156 (3)	0.018 (2)	−0.0303 (18)	0.067 (3)
Cl6	0.0665 (12)	0.0554 (12)	0.1098 (18)	0.0003 (9)	0.0135 (12)	−0.0212 (11)

### Geometric parameters (Å, °)

**Table:**

Ir1—C23	1.987 (5)	Ir2—N70	2.125 (5)
Ir1—C8	1.995 (6)	Ir2—O77	2.148 (4)
Ir1—N16	2.027 (5)	N40—C41	1.336 (6)
Ir1—N1	2.040 (4)	N40—C45	1.360 (7)
Ir1—N31	2.123 (5)	C41—C42	1.358 (8)
Ir1—O38	2.153 (4)	C41—H41	0.93
N1—C2	1.345 (7)	C42—C43	1.377 (8)
N1—C6	1.369 (7)	C42—H42	0.93
C2—C3	1.361 (8)	C43—C44	1.378 (7)
C2—H2	0.93	C43—C52	1.504 (8)
C3—C4	1.380 (8)	C44—C45	1.383 (7)
C3—H3	0.93	C44—H44	0.93
C4—C5	1.379 (9)	C45—C46	1.460 (7)
C4—C13	1.495 (8)	C46—C51	1.385 (8)
C5—C6	1.372 (8)	C46—C47	1.407 (8)
C5—H5	0.93	C47—C48	1.414 (7)
C6—C7	1.470 (8)	C48—C49	1.367 (9)
C7—C12	1.377 (9)	C48—H48	0.93
C7—C8	1.398 (8)	C49—C50	1.361 (9)
C8—C9	1.389 (8)	C49—F54	1.368 (6)
C9—C10	1.357 (10)	C50—C51	1.362 (8)
C9—H9	0.93	C50—H50	0.93
C10—C11	1.348 (11)	C51—F53	1.357 (7)
C10—F15	1.357 (8)	C52—H52A	0.96
C11—C12	1.361 (10)	C52—H52B	0.96
C11—H11	0.93	C52—H52C	0.96
C12—F14	1.351 (8)	N55—C56	1.347 (7)
C13—H13A	0.96	N55—C60	1.362 (7)
C13—H13B	0.96	C56—C57	1.364 (9)
C13—H13C	0.96	C56—H56	0.93
N16—C17	1.339 (7)	C57—C58	1.385 (9)
N16—C21	1.356 (7)	C57—H57	0.93
C17—C18	1.364 (9)	C58—C59	1.375 (8)
C17—H17	0.93	C58—C67	1.508 (8)
C18—C19	1.384 (9)	C59—C60	1.395 (8)
C18—H18	0.93	C59—H59	0.93
C19—C20	1.381 (9)	C60—C61	1.464 (7)
C19—C28	1.506 (8)	C61—C66	1.381 (8)
C20—C21	1.380 (8)	C61—C62	1.429 (7)
C20—H20	0.93	C62—C63	1.395 (7)
C21—C22	1.460 (8)	C63—C64	1.369 (8)
C22—C27	1.382 (8)	C63—H63	0.93
C22—C23	1.420 (8)	C64—F69	1.347 (6)
C23—C24	1.394 (8)	C64—C65	1.392 (8)
C24—C25	1.367 (8)	C65—C66	1.361 (8)
C24—H24	0.93	C65—H65	0.93
C25—F30	1.356 (6)	C66—F68	1.362 (6)
C25—C26	1.369 (9)	C67—H67A	0.96
C26—C27	1.370 (9)	C67—H67B	0.96
C26—H26	0.93	C67—H67C	0.96
C27—F29	1.346 (7)	N70—C71	1.343 (7)
C28—H28A	0.96	N70—C75	1.348 (7)
C28—H28B	0.96	C71—C72	1.366 (8)
C28—H28C	0.96	C71—H71	0.93
N31—C32	1.339 (7)	C72—C73	1.380 (8)
N31—C36	1.354 (7)	C72—H72	0.93
C32—C33	1.379 (8)	C73—C74	1.384 (9)
C32—H32	0.93	C73—H73	0.93
C33—C34	1.375 (8)	C74—C75	1.375 (8)
C33—H33	0.93	C74—H74	0.93
C34—C35	1.375 (9)	C75—C76	1.504 (8)
C34—H34	0.93	C76—O78	1.242 (7)
C35—C36	1.368 (8)	C76—O77	1.263 (7)
C35—H35	0.93	C79—Cl2	1.634 (10)
C36—C37	1.511 (8)	C79—Cl1	1.690 (10)
C37—O39	1.237 (7)	C79—Cl3	1.828 (10)
C37—O38	1.265 (7)	C79—H79	0.98
Ir2—C62	1.979 (5)	C83—Cl5	1.698 (10)
Ir2—C47	1.991 (5)	C83—Cl6	1.716 (8)
Ir2—N55	2.019 (4)	C83—Cl4	1.774 (10)
Ir2—N40	2.046 (4)	C83—H83	0.98
			
C23—Ir1—C8	86.8 (2)	C62—Ir2—N70	96.78 (18)
C23—Ir1—N16	80.4 (2)	C47—Ir2—N70	174.52 (18)
C8—Ir1—N16	93.4 (2)	N55—Ir2—N70	89.37 (18)
C23—Ir1—N1	95.6 (2)	N40—Ir2—N70	97.20 (17)
C8—Ir1—N1	80.2 (2)	C62—Ir2—O77	172.13 (18)
N16—Ir1—N1	172.69 (19)	C47—Ir2—O77	98.49 (18)
C23—Ir1—N31	97.73 (19)	N55—Ir2—O77	94.91 (17)
C8—Ir1—N31	174.22 (19)	N40—Ir2—O77	89.09 (16)
N16—Ir1—N31	90.89 (18)	N70—Ir2—O77	76.80 (16)
N1—Ir1—N31	95.75 (17)	C41—N40—C45	117.7 (4)
C23—Ir1—O38	172.21 (18)	C41—N40—Ir2	125.8 (3)
C8—Ir1—O38	99.14 (19)	C45—N40—Ir2	116.5 (3)
N16—Ir1—O38	94.13 (17)	N40—C41—C42	123.9 (5)
N1—Ir1—O38	90.39 (16)	N40—C41—H41	118.1
N31—Ir1—O38	76.70 (16)	C42—C41—H41	118.1
C2—N1—C6	118.1 (5)	C41—C42—C43	119.7 (5)
C2—N1—Ir1	125.5 (4)	C41—C42—H42	120.2
C6—N1—Ir1	116.4 (4)	C43—C42—H42	120.2
N1—C2—C3	123.2 (5)	C42—C43—C44	117.1 (5)
N1—C2—H2	118.4	C42—C43—C52	121.5 (5)
C3—C2—H2	118.4	C44—C43—C52	121.4 (5)
C2—C3—C4	119.8 (6)	C43—C44—C45	121.4 (5)
C2—C3—H3	120.1	C43—C44—H44	119.3
C4—C3—H3	120.1	C45—C44—H44	119.3
C5—C4—C3	117.2 (6)	N40—C45—C44	120.3 (5)
C5—C4—C13	121.4 (6)	N40—C45—C46	113.0 (5)
C3—C4—C13	121.4 (6)	C44—C45—C46	126.7 (5)
C6—C5—C4	121.8 (6)	C51—C46—C47	118.9 (5)
C6—C5—H5	119.1	C51—C46—C45	126.0 (5)
C4—C5—H5	119.1	C47—C46—C45	115.1 (5)
N1—C6—C5	120.0 (5)	C46—C47—C48	118.1 (5)
N1—C6—C7	112.5 (5)	C46—C47—Ir2	115.3 (4)
C5—C6—C7	127.5 (5)	C48—C47—Ir2	126.6 (4)
C12—C7—C8	118.9 (6)	C49—C48—C47	118.7 (5)
C12—C7—C6	125.5 (6)	C49—C48—H48	120.7
C8—C7—C6	115.6 (5)	C47—C48—H48	120.7
C9—C8—C7	117.6 (6)	C50—C49—C48	124.4 (5)
C9—C8—Ir1	127.4 (5)	C50—C49—F54	118.4 (6)
C7—C8—Ir1	115.0 (4)	C48—C49—F54	117.1 (6)
C10—C9—C8	120.0 (7)	C49—C50—C51	116.5 (5)
C10—C9—H9	120	C49—C50—H50	121.8
C8—C9—H9	120	C51—C50—H50	121.8
C11—C10—C9	123.7 (7)	F53—C51—C50	116.3 (5)
C11—C10—F15	117.7 (7)	F53—C51—C46	120.2 (5)
C9—C10—F15	118.5 (7)	C50—C51—C46	123.4 (6)
C10—C11—C12	116.4 (7)	C43—C52—H52A	109.5
C10—C11—H11	121.8	C43—C52—H52B	109.5
C12—C11—H11	121.8	H52A—C52—H52B	109.5
F14—C12—C11	117.0 (6)	C43—C52—H52C	109.5
F14—C12—C7	119.7 (6)	H52A—C52—H52C	109.5
C11—C12—C7	123.3 (7)	H52B—C52—H52C	109.5
C4—C13—H13A	109.5	C56—N55—C60	119.3 (5)
C4—C13—H13B	109.5	C56—N55—Ir2	122.9 (4)
H13A—C13—H13B	109.5	C60—N55—Ir2	117.5 (4)
C4—C13—H13C	109.5	N55—C56—C57	121.5 (6)
H13A—C13—H13C	109.5	N55—C56—H56	119.3
H13B—C13—H13C	109.5	C57—C56—H56	119.3
C17—N16—C21	119.6 (5)	C56—C57—C58	121.0 (6)
C17—N16—Ir1	123.3 (4)	C56—C57—H57	119.5
C21—N16—Ir1	116.9 (4)	C58—C57—H57	119.5
N16—C17—C18	122.0 (6)	C59—C58—C57	117.3 (6)
N16—C17—H17	119	C59—C58—C67	121.3 (6)
C18—C17—H17	119	C57—C58—C67	121.3 (6)
C17—C18—C19	120.0 (6)	C58—C59—C60	120.9 (6)
C17—C18—H18	120	C58—C59—H59	119.6
C19—C18—H18	120	C60—C59—H59	119.6
C20—C19—C18	117.5 (6)	N55—C60—C59	119.9 (5)
C20—C19—C28	121.6 (6)	N55—C60—C61	112.6 (5)
C18—C19—C28	120.9 (6)	C59—C60—C61	127.5 (5)
C21—C20—C19	121.1 (6)	C66—C61—C62	119.1 (5)
C21—C20—H20	119.5	C66—C61—C60	126.4 (5)
C19—C20—H20	119.5	C62—C61—C60	114.5 (5)
N16—C21—C20	119.8 (5)	C63—C62—C61	117.5 (5)
N16—C21—C22	112.9 (5)	C63—C62—Ir2	127.3 (4)
C20—C21—C22	127.3 (5)	C61—C62—Ir2	115.1 (4)
C27—C22—C23	118.6 (5)	C64—C63—C62	120.0 (5)
C27—C22—C21	126.3 (5)	C64—C63—H63	120
C23—C22—C21	115.1 (5)	C62—C63—H63	120
C24—C23—C22	118.3 (5)	F69—C64—C63	119.5 (5)
C24—C23—Ir1	127.1 (4)	F69—C64—C65	116.8 (5)
C22—C23—Ir1	114.6 (4)	C63—C64—C65	123.6 (5)
C25—C24—C23	119.6 (5)	C66—C65—C64	115.8 (5)
C25—C24—H24	120.2	C66—C65—H65	122.1
C23—C24—H24	120.2	C64—C65—H65	122.1
F30—C25—C24	118.8 (5)	C65—C66—F68	116.0 (5)
F30—C25—C26	117.5 (5)	C65—C66—C61	123.9 (5)
C24—C25—C26	123.7 (5)	F68—C66—C61	120.1 (5)
C25—C26—C27	116.5 (5)	C58—C67—H67A	109.5
C25—C26—H26	121.7	C58—C67—H67B	109.5
C27—C26—H26	121.7	H67A—C67—H67B	109.5
F29—C27—C26	116.5 (5)	C58—C67—H67C	109.5
F29—C27—C22	120.2 (5)	H67A—C67—H67C	109.5
C26—C27—C22	123.3 (6)	H67B—C67—H67C	109.5
C19—C28—H28A	109.5	C71—N70—C75	118.6 (5)
C19—C28—H28B	109.5	C71—N70—Ir2	126.9 (4)
H28A—C28—H28B	109.5	C75—N70—Ir2	114.4 (4)
C19—C28—H28C	109.5	N70—C71—C72	121.8 (5)
H28A—C28—H28C	109.5	N70—C71—H71	119.1
H28B—C28—H28C	109.5	C72—C71—H71	119.1
C32—N31—C36	118.8 (5)	C71—C72—C73	120.0 (6)
C32—N31—Ir1	126.6 (4)	C71—C72—H72	120
C36—N31—Ir1	114.6 (4)	C73—C72—H72	120
N31—C32—C33	121.3 (5)	C72—C73—C74	118.3 (6)
N31—C32—H32	119.3	C72—C73—H73	120.8
C33—C32—H32	119.3	C74—C73—H73	120.8
C34—C33—C32	119.9 (6)	C75—C74—C73	119.2 (5)
C34—C33—H33	120	C75—C74—H74	120.4
C32—C33—H33	120	C73—C74—H74	120.4
C33—C34—C35	118.5 (6)	N70—C75—C74	122.0 (5)
C33—C34—H34	120.8	N70—C75—C76	115.7 (5)
C35—C34—H34	120.8	C74—C75—C76	122.3 (5)
C36—C35—C34	119.7 (6)	O78—C76—O77	125.3 (6)
C36—C35—H35	120.2	O78—C76—C75	118.2 (5)
C34—C35—H35	120.2	O77—C76—C75	116.4 (5)
N31—C36—C35	121.8 (5)	C76—O77—Ir2	116.4 (3)
N31—C36—C37	115.6 (5)	Cl2—C79—Cl1	117.3 (6)
C35—C36—C37	122.6 (5)	Cl2—C79—Cl3	108.0 (5)
O39—C37—O38	125.5 (5)	Cl1—C79—Cl3	107.3 (5)
O39—C37—C36	118.4 (5)	Cl2—C79—H79	108
O38—C37—C36	116.1 (5)	Cl1—C79—H79	108
C37—O38—Ir1	116.8 (3)	Cl3—C79—H79	108
C62—Ir2—C47	88.1 (2)	Cl5—C83—Cl6	111.0 (5)
C62—Ir2—N55	80.3 (2)	Cl5—C83—Cl4	111.4 (5)
C47—Ir2—N55	93.9 (2)	Cl6—C83—Cl4	108.7 (5)
C62—Ir2—N40	96.29 (19)	Cl5—C83—H83	108.6
C47—Ir2—N40	79.8 (2)	Cl6—C83—H83	108.6
N55—Ir2—N40	172.96 (17)	Cl4—C83—H83	108.6
